# The diagnosis and treatment of deltoid ligament lesions in supination–external rotation ankle fractures: a review

**DOI:** 10.1007/s11751-012-0140-9

**Published:** 2012-07-06

**Authors:** Sjoerd A. S. Stufkens, Michel P. J. van den Bekerom, Markus Knupp, Beat Hintermann, C. Niek van Dijk

**Affiliations:** 1 Department of Orthopaedic Surgery, Academic Medical Centre, P.O. Box 22660, 1100 DD Amsterdam, The Netherlands; 2 Department of Orthopaedic Surgery, Kantonsspital Liestal, Rheinstrasse 26, 4410 Liestal, Switzerland

**Keywords:** Ankle fracture, Deltoid ligament, Stability, Diagnosis, Treatment

## Abstract

The supination–external rotation or Weber B type fracture exists as a stable and an unstable type. The unstable type has a medial malleolus fracture or deltoid ligament lesion in addition to a fibular fracture. The consensus is the unstable type and best treated by open reduction and internal fixation. The diagnostic process for a medial ligament lesion has been well investigated but there is no consensus as to the best method of assessment. The number of deltoid ruptures as a result of an external rotation mechanism is higher than previously believed. The derivation of the injury mechanism could provide information of the likely ligamentous lesion in several fracture patterns. The use of the Lauge-Hansen classification system in the assessment of the initial X-ray images can be helpful in predicting the involvement of the deltoid ligament but the reliability in terms of sensitivity and specificity is unknown. Clinical examination, stress radiography, magnetic resonance imaging, arthroscopy, and ultrasonography have been used to investigate medial collateral integrity in cases of ankle fractures. None of these has shown to possess the combination of being cost-effective, reliable and easy to use; currently gravity stress radiography is favoured and, in cases of doubt, arthroscopy could be of value. There is a disagreement as to the benefit of repair by suture of the deltoid ligament in cases of an acute rupture in combination with a lateral malleolar fracture. There is no evidence found for suturing but exploration is thought to be beneficial in case of interposition of medial structures.

## Introduction

Supination–external rotation (SE) fractures, also known as Weber B type fractures, are the most common ankle fractures and account for as many as 80 % of all ankle fractures [23, 24, 49, 55, 61, 69, 131]. A decision for operative or nonoperative treatment is based on the stability of the ankle as operatively managed unstable fractures have a better outcome than those treated conservatively [4, 6, 9, 17, 73, 102]. Medial instability associated with a lateral malleolar fracture can result from a medial malleolar fracture, a deltoid ligament lesion or a combination of osseous and ligamentous lesions. The diagnosis of deltoid ligament lesions in SE fractures has limitations. Several authors have reported the possibility of unrecognized unstable fractures in their series of stable fractures (which are often treated conservatively), negatively influencing the outcome [4, 61, 103, 131]. Differentiation of unstable and stable types is therefore important. In this review of the deltoid ligament in SE ankle fractures, we provide an overview of present knowledge on this topic as reported in the literature and based on the experience of two experienced foot and ankle surgeons (BH and CNvD). We focus on the SE type of ankle fractures as they represent the main body of ankle fractures and present a diagnostic challenge. This review is to communicate the need for continued research for diagnostic methods and treatment strategies regarding the injury of this ligament. Levels of evidence were applied to the individual studies reviewed and grades were applied to the recommendations for clinical practice (Table 1).

**Table 1:** Level of evidence and grades of recommendation

*Level of evidence*
Level I: high-quality prospective randomized clinical trial
Level II: prospective comparative study
Level III: retrospective case–control study
Level IV: case series
Level V: expert opinion
*Grades of recommendation* (given to various treatment options based on level of evidence supporting that treatment)
*Grade A* treatment options are supported by strong evidence (consistent with level I or II studies)
*Grade B* treatment options are supported by fair evidence (consistent with level III or IV studies)
*Grade C* treatment options are supported by either conflicting or poor quality evidence (level IV studies)
*Grade I* when insufficient evidence exists to make a recommendation

## Anatomy

**Fig. 1:**
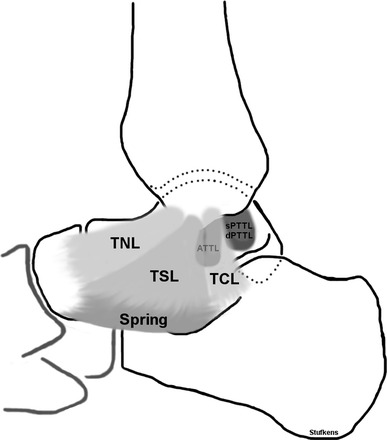
Anatomic configuration of the deltoid ligament. *TNL* tibionavicular ligament, *TSL* tibiospring ligament, *Spring* spring ligament, *TCL* tibiocalcaneal ligament, *ATTL* anterior tibiotalar ligament, *sPTTL* superficial posterior tibiotalar ligament, *dPTTL* deep posterior tibiotalar ligament

The general bony anatomy of the ankle joint is well known. The medial malleolus has two colliculi divided by a groove. On the posterolateral side, the posterior tibial tendon (PTT) and the flexor digitorum longus (FDL) pass. The deltoid ligament is attached to both colliculi proximally and has several insertions distally on the navicular, talus and calcaneus and onto the spring ligament. The narrow proximal anchoring and multiple distal attachments give the ligament, its typical shape and its name. The first anatomical division is between superficial and deep layers of the ligament. The superficial fibres originate on the anterior colliculus and cross two joints (tibiotalar and talocalcaneal), whereas the deep part, originating in the groove between and on the posterior colliculus, only bridges the tibiotalar joint. Historically, other authors have described from three to six differing anatomical divisions [13, 22, 44, 58, 97, 116]. In our opinion, the deltoid ligament is comprised of six different parts according to different functional properties (see Fig. 1). Superficial and anterior are the tibionavicular (TNL), the tibiospring (TSL) and the tibiocalcaneal (TCL) ligaments. The deep layer consists of the superficial posterior (sPTTL), deep posterior (dPTTL) and anterior tibiotalar (ATTL) ligaments [13, 44, 88]. There can be individual differences; the TNL is considered by some to be a thickened fibrous part of the anterior ankle capsule, rather than a separate ligament [13]. The dPTTL appears on magnetic resonance imaging (MRI) and in anatomical dissection as thickest and bridges the posterior colliculus and the medial tubercle of the talus. The TSL is the component of the deltoid ligament without two bony attachments. It originates from the anterior colliculus and fans out to the plantar calcaneonavicular or spring ligament thereby forming a functional unit with this ligament. It appears on MRI as the second largest component [58]. In anatomical preparations, however, the TCL is recognized as being at least as thick as the dPTTL [13]. When Mengiardi et al. [13, 82] evaluated the visibility and signal intensity characteristics of the deltoid ligament on MRI in asymptomatic volunteers, the dPTTL and TSL were always visible. The ATTL and TNL were only seen in about half of the subjects.

## Biomechanics of the deltoid ligament

The deltoid ligament is thought to have a dual function; to provide medial stability to the tibiotalar joint and to transfer forces between tibia and tarsus [47, 50, 114]. The primary function of the deltoid ligament is the firm fixation of the tibia above the talus and to restrict the tendency of the talus to shift into a valgus position, to translate anterolaterally or to externally rotate. The intact deltoid ligament prevents the talus shifting more than 2 mm laterally, even if the lateral structures are not in place [22, 42, 64, 97, 108]. Normal movement of the talus in the mortise is possible in all three planes. The normal range of motion is described variously: plantar flexion is reported to exceed dorsiflexion by 4–5 times or by up to 80 % [12, 48, 65, 108]; at maximum plantar flexion of the foot, internal rotation of 1.9° of the talus is seen, whereas at maximum dorsiflexion 7.2° of external rotation [83]. Adduction and abduction with intact ligaments are widely disputed and range from 5° symmetrically to some extreme values [10, 66, 108, 112]. Internal and external rotation have been reported to range from 14 to 24° [56, 79, 108].

Cutting of the deltoid ligament has been performed by several authors in order to investigate its function [39, 93, 108]. Severe instability is reported when cutting the entire ligament but a surprising degree of stability is found remaining when cutting only the superficial part of the deltoid ligament. With the deep part still intact, only 4–7° of external rotation of the talus was possible [79, 108]. In the absence of a medial injury, a complete fibular osteotomy does not cause abnormal motion of the ankle [83, 111]. The ATTL together with the anterior talofibular ligament on the lateral side is thought to restrict forward translation of the talus. However, some authors state that the ATTL has no independent function and that the lateral ligament mainly restricts plantar flexion [108]. According to Dehne and Dias [27, 30], the posterior tibiotalar ligament restricts internal rotation of the talus solely by means of its deep fibres. However, these authors have not performed isolated sectioning of these fibres. In a study of injuries to the different ankle ligaments performed by Rasmussen, it was found that cutting of both the TCL and the ATTL hardly affected talar movement in any direction [108].

There is an agreement between radiological and anatomic studies over the strength of the different components of the deltoid ligament. The dPTTL appears to be the strongest followed by the TSL. The TCL and TNL are weaker than the latter [58, 97, 108, 119]. In addition, there is interlacing of the TSL and the TNL. This spring ligament complex supports the talar head medially and stabilizes the entire talocalcaneonavicular joint. Hintermann also suggests a relationship between laxity of this ligament complex and medial ankle instability [51].

The weakness of in vitro studies is many authors have used nonstandardized forces to induce movements of the separate structures in the ankle joint. The results of these biomechanical studies are to be interpreted with caution as the cadaver does not bear weight and the ligaments may behave differently in vivo.

## Mechanism of trauma

**Fig. 2:**
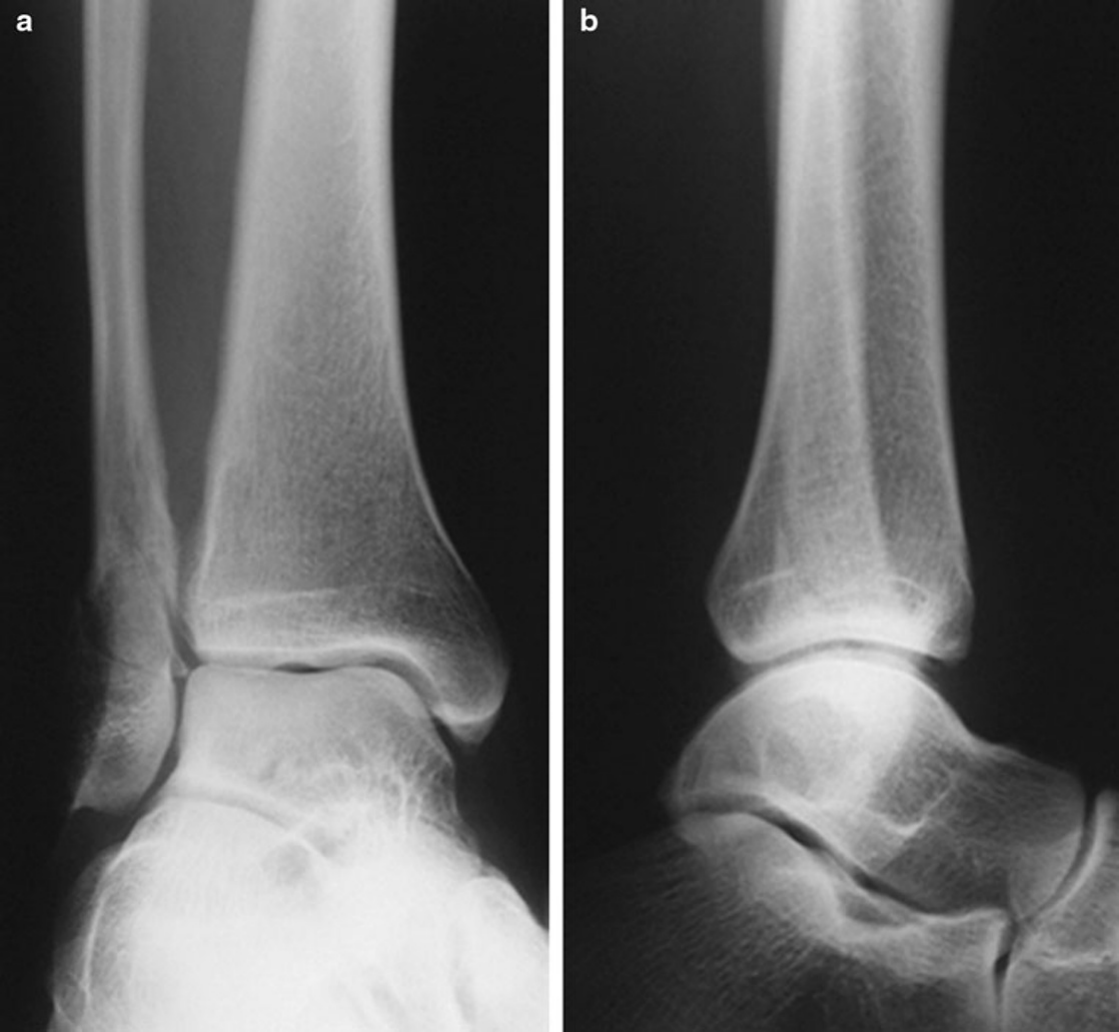
AP and lateral radiographic images of a SE-2 fracture, consisting of a spiral or oblique fibula fracture at the level of the syndesmosis

**Fig. 3:**
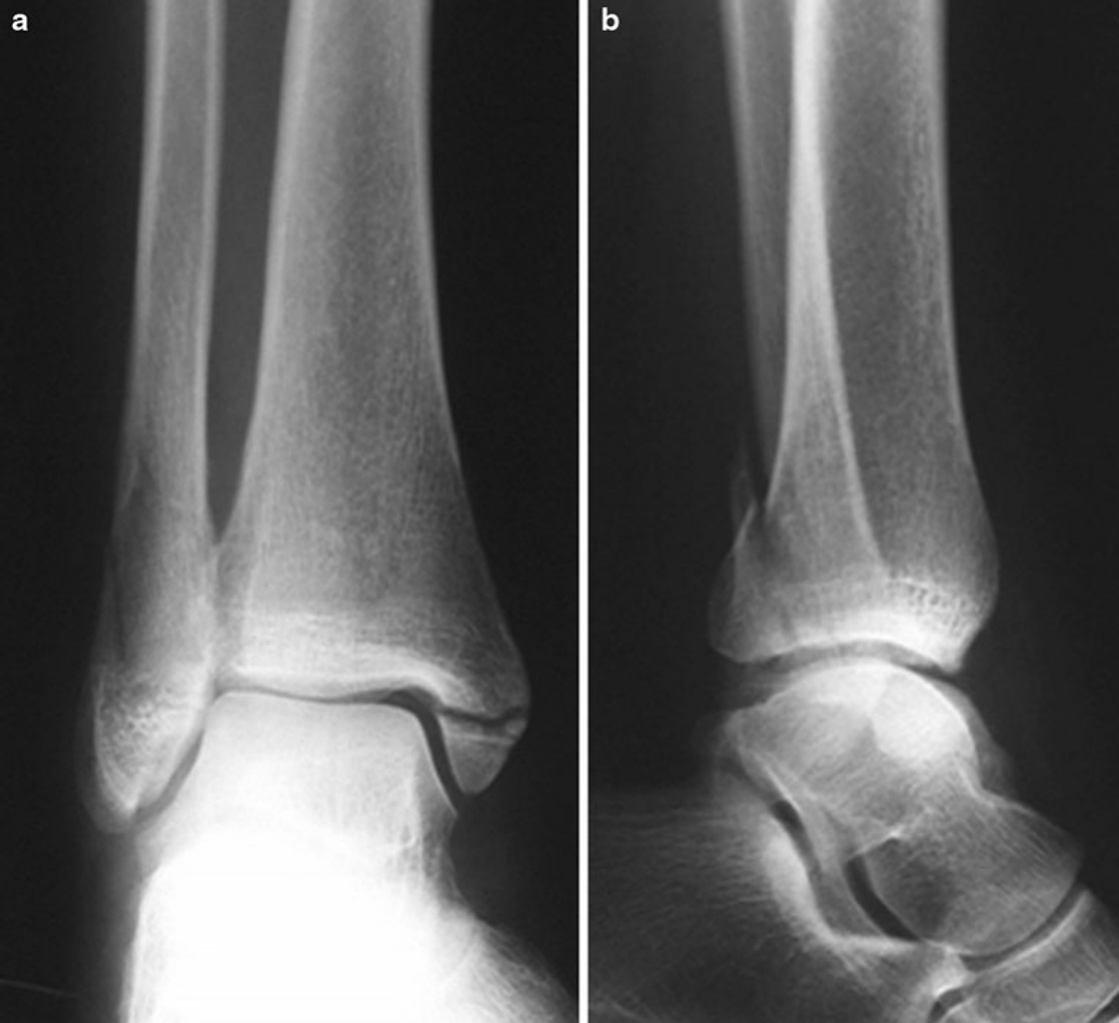
AP and lateral radiographic images of a SE-4 fracture consisting of a spiral or oblique fracture laterally and a transverse medial malleolar (avulsion) fracture

**Fig. 4:**
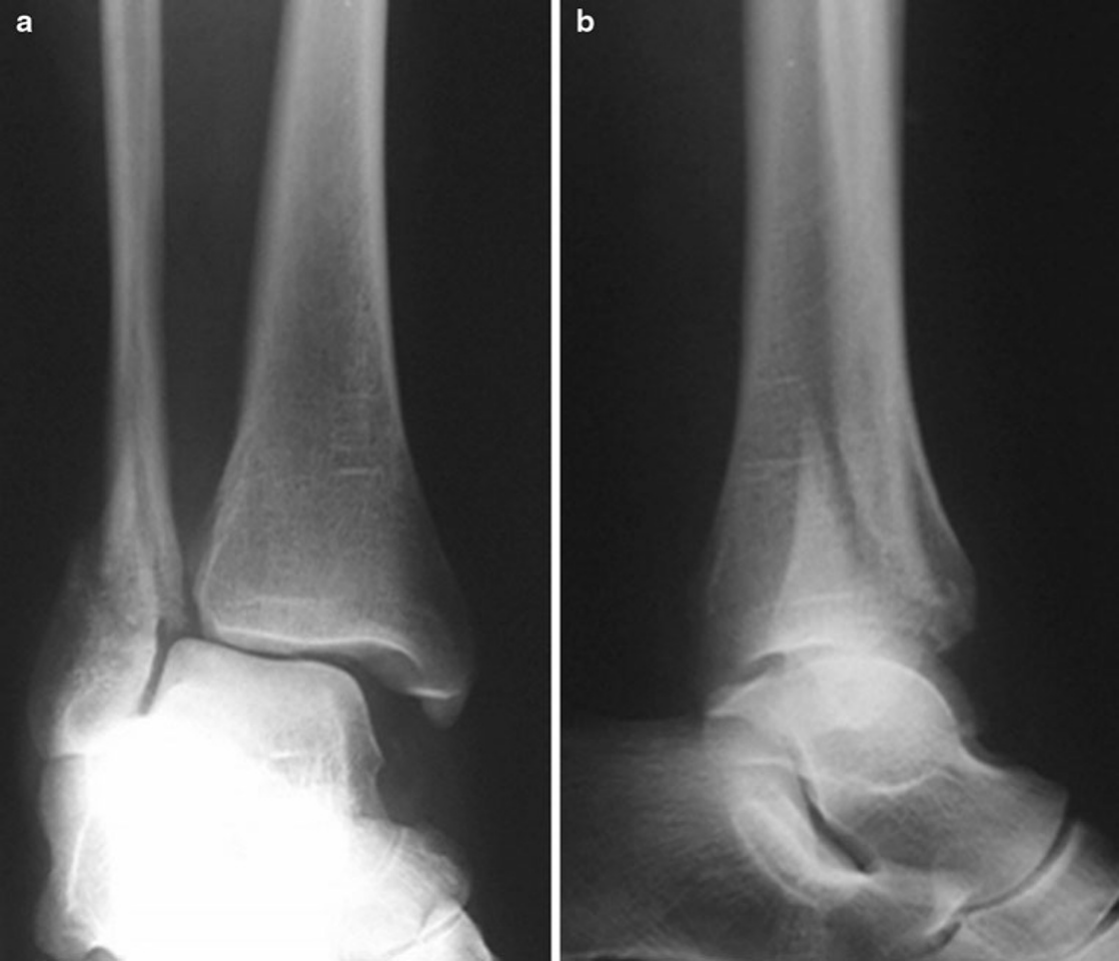
AP and lateral radiographic images of a SE-4 fracture consisting of a spiral or oblique fracture laterally and a deep deltoid rupture, allowing a talar shift (resulting in widening of the medial clear space)

The main causes of deltoid ligament lesions are pronation or rotation movements of the hindfoot [4, 53, 61, 64, 69, 72, 131]. The first systematic investigation of ankle fracture patterns and the accompanying injury to ankle joint ligaments was done by Lauge-Hansen. Although several of the proposed injury mechanisms and the height of the fibular fracture in SE fractures have been disputed by some authors, many studies are based on his work and his terminology has become widely used [40, 54, 69, 85, 98, 127]. His system of fracture and ligament injury pattern is based on cadaver experiments. Lauge-Hansen simulated several rotational, abduction and adduction movements of the lower leg with regard to a fixed foot in pronation or in supination. SE rotation is the mechanism that causes approximately 80 % of all ankle fractures. Lauge-Hansen found that in stage one, a rupture or avulsion of the anterior tibiofibular ligament occurs. The deltoid ligament is lax since the position of the foot is in supination. Further external rotation of the foot increases the pressure of the talus against the fibula results in a twisting motion of the fibula around its longitudinal axis, producing the typical spiral (Weber B) fracture at the level of the syndesmosis (see Fig. 2). In this second stage, the deltoid ligament is still lax since the foot is still supinated. The interosseous transverse ligament, interosseous membrane, posterior syndesmosis and deltoid ligament remain intact at this stage. When the external rotation is continued, the talus is subluxed and the hindfoot adopts a valgus position. The foot cannot maintain the supinated position which becomes neutral and now moves into a pronated position but still without rupture of the deltoid ligament. During this movement, the tip of the fractured fibula and the talus can collide with the posterior tibial tubercle, resulting in a splitting off of a triangular shaped piece also known as Volkmann’s fracture. The posterior tibiofibular ligaments are very strong and rupture is uncommon [72, 127]. In the original experiments, a posterior malleolar fracture or posterior talofibular ligament rupture was named stage three. When more external rotation was performed, a fracture of the medial malleolus resulted (see Fig. 3). In his first report, Lauge-Hansen did not describe deltoid ruptures from this (end-stage) grade four SE fracture. In later publications, he stated that medial malleolar fractures could be replaced by deltoid ligament injury which completed his system of injury patterns arising from SE rotation forces (see Fig. 4) [63, 64]. Other authors deduced from the described mechanism that in stage four avulsion fractures should occur at the same rate as deltoid ligament ruptures [4, 20, 69, 123, 131]. Moreover, Rasmussen found that especially the deep portions of the deltoid ligament, which are thought to be the main stabilizers, could rupture in external rotation while the superficial components remain intact [108].

**Fig. 5:**
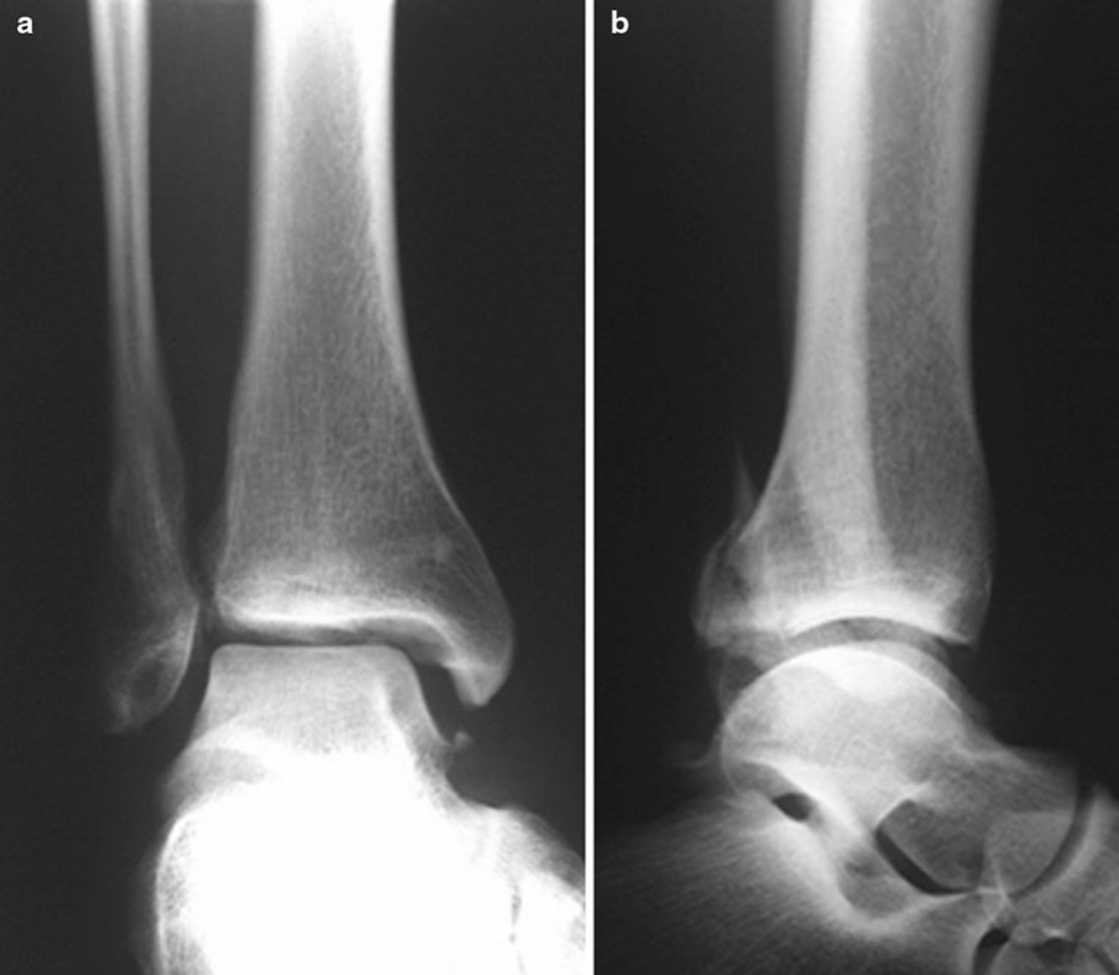
AP and lateral radiographic images of a SE-4 fracture consisting of a spiral or oblique fracture laterally with a combination of an avulsion fracture medially. There may also be a deep deltoid rupture. When in doubt, medial integrity could be tested by gravity stress radiography

Approximately, a quarter of patients with stage four SE fractures are thought to suffer an avulsion fracture medially and a rupture of one of the components of the deltoid ligament (Fig. 5) [117]. In bimalleolar fractures, the medial injury may appear to be this osseous avulsion only with the deltoid ligament left intact on the displaced fragment but this injury may also be a combination of ligamentous and osseous injury with disruption of the deep portion of the deltoid ligament. As reported by several authors, there should be awareness of the possibility of a deltoid rupture in combination with a medial fracture [94, 117]. The superficial component of the deltoid ligament is thin and weaker than the deep part and is under tension during external rotation of the ankle when the foot is in plantar flexion. Therefore, fixation of small anterior fractures of the medial malleolus, to which only the superficial portion of the ligament attaches, may not be sufficient to restore medial stability [40, 94, 125]. In 60–70 % of the avulsion fractures of the posterior colliculus, the strong posterior tibiotalar ligament remains intact and attached to the fractured fragment, while the other weaker components are torn [58, 117, 119].

The mechanism underlying SE and pronation–external rotation (PE) fractures is similar. The difference is the position of the foot at the moment of external rotation. With a foot in pronation, there is initial tension on the medial structures. A lateral fracture resulting from PE is unstable for there is always a medial fracture or deltoid rupture. This has been questioned by authors who report observing high fibular fractures without injury at the medial side [40, 85]. The frequency of injury to the deltoid ligament in SE fractures is higher than previously expected and ranges from 20 to 50 % [49, 69]. These figures may underestimate the true frequency due to lack of diagnostic reliability.

## Diagnosis

The Lauge-Hansen classification has the additional advantage of taking ligamentous injuries into account. The comprehensiveness of the system does make it more difficult to use than the Weber classification [64, 69, 131]. Thorough knowledge of ankle anatomy and subgroups of the Lauge-Hansen system are required for its application but, although precision can be improved by teaching, some studies have shown that the system cannot be applied consistently with only poor to fair inter-observer reliability [2, 85, 87, 92, 110, 130]. The problem of inconsistent application of the Lauge-Hansen scheme is compounded by fractures patterns that escape this classification system [19, 40]. Some fractures considered stable by the Lauge-Hansen classification may require careful examination to rule out deep deltoid injury [19]. Therefore, the diagnostic value of the Lauge-Hansen classification for ligamentous injuries in SE fractures seems limited. Although the Lauge-Hansen system is not infallible, 91.6 % of the fractures in the study of Schuberth et al. [117] that were classifiable according to the scheme demonstrated the expected deltoid ligament findings. The problem in SE fractures is the ‘invisible medial injury’ [121]. The decision to treat a seemingly stable SE stage two fracture conservatively, without accurate assessment of deltoid ligament injury, may predispose a patient to early posttraumatic osteoarthritis [5, 68, 101, 117, 132]. Rupture of the deep deltoid ligament combined with a displaced lateral malleolar fracture is the biomechanical equivalent of a bimalleolar fracture and is best treated with open reduction and internal fixation of the fibula to restore ankle mortise anatomy [21, 108, 117].

A recent systematic review of the modalities for evaluation of the integrity of the deltoid ligament in patients with SE ankle fractures was published by van den Bekerom et al. [8] (level I evidence). Many orthopaedic surgeons rely on clinical signs such as ecchymosis, swelling and tenderness to evaluate integrity of the medial structures [3, 16, 35, 74, 97, 98, 104]. Similarly, in lateral ligament injuries, clinical evaluation has been proven to be of great value; additional (imaging) investigation has shown little or no added contribution to accurately make the diagnosis [32]. A review in a publication from the American Academy of Orthopaedic Surgeons supports the use of medial tenderness as a predictor of deep deltoid disruption in SE type ankle fractures [7]. Despite this, the current literature cautions against clinical features of the injured ankle as adequate predictors of medial stability of the ankle joint (based on level III and IV evidence) [26, 36, 78]. When these clinical symptoms are present, it may be likely that there is a soft-tissue injury. This injury could consist of only the superficial deltoid ligaments with intact deep structures. The superficial ligaments deliver little contribution to medial stability of the ankle and, like the stronger deep component, can also be injured by means of a rotational mechanism [78, 84, 108].

As the initial radiographs of an ankle injury with an isolated distal fibular fracture at the level of the syndesmosis may be inconclusive, a stress radiograph has been recommended to determine the integrity of the medial clear space (based on level III and IV evidence) [36, 62, 67, 78, 127]. The medial clear space is measured from the superior-medial aspect of the talus to the superior-medial corner of the tibial plafond. External rotation stress radiographs, as described by Pankovich, are considered the gold standard but this test has its shortcomings and has never been validated (level IV evidence) [44, 78, 97, 98, 124]. Tornetta stated that these tests are the gold standard for subluxation as an indirect measurement of deltoid injury or deltoid insufficiency [125]. The reported amount of widening of the medial clear space as indicative for a positive external rotation stress test or gravity stress test varies [19, 25, 36, 41, 44, 46, 57, 75, 78, 86, 99, 102, 117]. Normal values are reported to vary from 1 to 5 mm [19, 25, 44, 46, 75]. A medial clear space of more than 4 mm, with that value being at least 1 mm greater than the superior tibiotalar space, is accepted to represent a deep deltoid ligament rupture (based on level III and IV evidence) [3, 35, 36, 41, 45, 78, 87, 102]. In a cadaver study, transection of the superficial deltoid ligament alone did not cause medial clear space widening, even in the presence of a fibular fracture [86]. However, an intact superficial part, and a negative abduction stress test, does not guarantee an intact deep ligament [108]. The direction of rotational stress applied to the foot has a greater effect on medial clear space in predicting deep deltoid ligament status than does the amount of ankle flexion. Stress radiographs obtained with the foot in dorsiflexion with addition of external rotation were most predictive of deep deltoid ligament disruption after distal fibular fracture [99]. The amount of applied force necessary when performing an external rotation stress radiograph is not well defined. Xenos recommends 5 Newton metre, McConnell and Park recommend 8 pounds and Tornetta used 20 pounds [25, 78, 99, 125]. Patients may experience pain during an ankle stress test which could then increase resistive muscle forces. This could limit the amount of rotation possible in the injured ankle; therefore, these tests are only well tolerated with the use of analgesics, narcotics or under general anaesthesia [41]. To solve this problem Michelson proposed a gravity stress test [86]. There was no significant difference between the gravity and manual stress radiograph with regard to mean medial clear space or talar shift measured in association with either fracture pattern [41]. The visual analogue pain score indicated that patients perceived more discomfort while being examined with manual stress applied compared to gravity testing (level III evidence). The main limitation of the gravity stress radiograph is the inability to control dorsiflexion and plantar flexion. However, this technique involves less radiation exposure to the physician and can be performed by assistant radiographers. The use of weight bearing radiographs as proposed by Weber et al. [128] is an easy, pain-free, safe and reliable method to exclude the need for operative treatment with excellent clinical outcome in the majority of the patients seen at latest follow-up. Further studies are required concerning this type of radiograph, because at last follow-up, the patients were only interviewed by phone only and no radiographs taken for final assessment. Asymptomatic ankle arthritis, ankle instability, or poor range of motion of the hindfoot joints might have been missed in this study.

Arthroscopy has been used to assess cartilage lesions and ligamentous damage in acute ankle trauma [31, 33, 49, 71]. Schuberth et al. [117] compared deep deltoid ligament integrity as seen with arthroscopy with corresponding medial clear space measurements in a clinical setting. They concluded that displaced SE fractures in patients with medial ankle tenderness, but without overt widening of the medial clear space on injury radiographs require careful attention because the integrity cannot be reliably predicted by injury radiographs. Damage to the ligaments cannot always be identified by arthroscopy. Hintermann reported that only 84.4 % of deltoid ligaments could be seen on arthroscopy directly after trauma and superficial components cannot be seen at all [49, 117].

Magnetic resonance imaging (MRI) may help in determining deltoid ligament integrity after trauma and for individual cases in which doubt about joint stability and soft-tissue integrity exists [40, 52, 118]. In a preliminary report, Koval et al. [60] concluded that medial clear space measurements on manual stress radiographic testing did not correlate with deep deltoid rupture on MRI (level IV evidence). These conclusions should be interpreted with caution because of the incomplete and short-term follow-up in their study. Clearly, there are limitations in its practicality because of cost and convenience.

Ultrasound imaging is often considered as a complementary modality to MRI. Modern ultrasound techniques like 3-D rendering have become competitive. The major advantages of ultrasound include dynamic evaluation of structures, low cost and wide availability. The main disadvantage is a high degree of operator dependency. In general, the cost-effectiveness of ultrasound could justify its use as a first-line examination technique [100, 109]. The deltoid ligaments are best visualized on sonograms when the hindfoot is turned laterally and the ankle is in dorsiflexion. This makes ultrasound investigation in ankle fractures difficult as when compared to investigating the ligaments in ankle sprain. In the acute setting of a ruptured deltoid, an anechoic zone crossing the ligament can be seen but also oedema, ecchymosis and avulsions of the bony insertion. On the lateral side of the ankle joint, sonography has been proven to correctly diagnose ligamentous lesions with accuracy as high as 87–100 % (level IV evidence) [14, 37, 38, 89]. Sonography, while useful for depicting and studying the integrity of the medial collateral ankle ligaments, has yet to be proven for detecting deltoid ruptures sustained in ankle fractures. Several authors advocate further research in the different imaging modalities [14, 18, 43, 90, 91, 107, 115].

## Treatment

In 1987, Baird and Jackson performed a review of the literature on the most appropriate treatment of ankle injuries in which the deltoid ligament is ruptured and the fibula is fractured at the level of the syndesmosis. Based on the premise that the ruptured ends of the deltoid ligament retract and are not apposed and that disrupted ligaments heal better when they are surgically approximated, they found twelve articles, which advocated surgical repair of the ligament in conjunction with reduction of the fibular fracture [1, 15, 22, 34, 59, 95, 96, 105, 106, 113, 120, 131]. However, nine other articles reported adequate results without surgical repair of the deltoid ligament [11, 28, 29, 63, 76, 80, 81, 121, 129, 130]. These depended on restoration of the normal osteoligamentous anatomy of the lateral structures of the ankle joint to achieve stability of the ankle. As the primary objective of these studies was not to evaluate the need for deltoid reconstruction, these studies had a limited number of patients. Moreover, there were different objective and subjective outcome measurements and it was difficult to reconcile the validity of contradictory viewpoints. In their own results (level IV evidence) of three sutured deltoid ligaments, two had poor results but these two ruptures were the result of a PE fracture, while the repaired ligament after SE type fracture had an excellent outcome [3]. A typical example of a brief mention in treatment of the deltoid ligament was reported by Lindsjö in an otherwise outstanding follow-up study of 327 ankle fractures: ‘The deltoid ligaments was sutured to similar extents in the two result groups “excellent to good” and “acceptable to poor”. Injuries to these ligaments do not appear to have been a discriminating factor of importance in this material’ [70].

We found only six publications in which the need for exploration and suturing the deltoid ligament after ankle fractures was the primary question (Table 2) [3, 45, 77, 122, 126, 133]. Although these studies are different in design and have different inclusion criteria, they have similar conclusions (based on level II–IV evidence). These studies show that in the event of an adequate reduction in the fractured fibula and normalization of the medial clear space, it is not necessary to explore the medial clear space and to reconstruct the deltoid ligament. Only if there is interposition on the medial side after adequate reduction in the fibular fracture is an exploration of the medial clear space required. However, in all six articles, there was not a single patient in which exploration was needed. Theoretically, soft tissue, scar tissue, ligament remnants, or chondral fragments may be interposed between the talus and the medial malleolus. If this is the case, they should be removed to enable an adequate reduction.

**Table 2:** Clinical studies evaluating suturing of the deltoid ligament after ankle fractures

Author	Study level	Number of patients treated	Type of injury	Number of patients available for follow-up	Mean follow-up (months)	Sutured	Outcome	Not sutured	Outcome	Conclusion
Baird and Jackson [3]	IV	70	Distal fibular fracture and disruption of the deltoid ligament	24 (13 SE#, 11 PE#)	36	3	1 SE# excellent, 2 PE# poor	21	8 SE# excellent, 5 PE# excellent, 3 SE# good, 3 PE# good, 1 SE# fair, 1 PE# poor	90 % of the nonrepaired ligaments had a good or excellent result. Only if the medial clear space remains widened after fracture reduction does the medial side need to be explored
Harper [45]	IV	42	Fracture dislocations of the ankle	36 (18 SE#, 15 PE#, 2 maisonneuve, 1 syndesmotic diastasis	30	0	–	36	12 SE# good, 4 SE# fair, 2 SE# poor, 14 PE# good, 1 PE# poor, 1 maisonneuve good, 1 maisonneuve poor, 1 diastasis good	The deltoid ligament will heal sufficiently with nonoperative treatment, provided that the medial joint space is maintained in a reduced position
Zeegers and van der Werken [133]	IV	28	Ankle fracture associated with a ruptured deltoid ligament	28 (12 SE#, 10 PE#, 6 PA#)	18	0	–	28	20 patients (very) good, 8 patients poor	After anatomical reconstruction of the lateral malleolus with perfect congruity of the ankle mortise there is no need to explore and suture the ruptured deltoid ligament
Strömsöe et al. [122]	II	50	Weber B and C types and a ruptured deltoid ligament	50 (30 Weber B, 20 Weber C)	17	25	No differences between groups	25	No differences between groups	A ruptured deltoid can be left unexplored. Operating time is reduced and the skin over the medial malleolus is left untouched
Maynou et al. [77]	III	44	Ankle fractures with deltoid ligament rupture	44 (7 OCD and 2 malreductions) were evaluated separately	56	18	2 medial instability	17	2 medial instability, more ossifications of the deltoid (*p* = 0.013), 1 posttraumatic osteoarthritis	Repair of the deltoid ligament is unnecessary if the internal fixation of the fibula achieves an anatomical reconstrucion of the mortise
Tourne et al. [126]	IV	48	Weber A, B and C fractures with a ruptured medial collateral ligament	33	27	0	–	33	82.5 % excellent and good, 73 % normal Rx, 15 % anterior impingement, 12 % deltoid calcifications	Suggestion to leave the ligament tears unexplored (medial, tibiofibular, and syndesmotic)

## Conclusion and recommendations

There have been many studies examining the diagnosis and treatment of SE type ankle fractures. In spite of common agreement on treating unstable fractures with open reduction and internal fixation, there have been reports of unsatisfying results with conservative treatment of seemingly stable fractures. The Weber classification does not take the status of ligaments into account whereas the Lauge-Hansen classification does. In SE type 2 fractures, the deltoid ligament is intact, but SE type 4 represents an unstable configuration. In case of tibiotalar displacement of more than four millimetres, there is no problem with making the diagnosis but in cases where the X-ray shows no displacement there still can be a deltoid ligament rupture. The question remains as to which diagnostic tools are the best at examining the integrity of deep portion of the deltoid ligament.

- The gravity stress radiograph has provided the best results in detection of deltoid ligament rupture in patients with SE ankle fractures.
- A medial clear space of over four millimetres seen after fibular fracture, with that value being at least one millimetre greater than the superior tibiotalar space, is a value that is accepted to represent a ruptured deep deltoid ligament.
- Other diagnostic criteria, such as pain over the deltoid ligament, swelling, ecchymosis, or combinations thereof have not shown sufficient sensitivity and specificity to rule out instability of the ankle joint, and further investigation is therefore warranted.
- Theoretically, ultrasound examination of the deltoid region has potential. Ultrasonography is, however, a dynamic investigation and requires experienced hands. Further studies comparing combinations of different diagnostic (imaging) modalities could improve inter- and intra-observer reliability.
- The treatment of deltoid ligament lesions (exploration and reconstruction of the deltoid ligament) is only necessary if there is interposition on the medial side after adequate reduction of the fibular fracture.
- When the fibula fracture is adequately reduced and the medial clear space has returned to its normal width there is no indication to perform an exploration.
- In cases of doubt, arthroscopy could be of assistance to determine interposition when the medial clear space remains wide after proper reduction.
